# Double whammy: Acute suppurative appendicitis on top of Meckel's diverticulum

**DOI:** 10.1016/j.radcr.2024.12.031

**Published:** 2025-01-15

**Authors:** Hosam Amir, Hadeer Hafez, Tibyan Basheer, Mohamed Abdelsalam

**Affiliations:** a Surgery department, Faculty of medicine, Misr university for science and technology, Giza 12566, Egypt; b Faculty of medicine, October 6th university, Giza 12566, Egypt; c Faculty of medicine, AlMugtaribeen university, Khartoum 12217, Sudan; d Faculty of medicine, Misr university for science and technology, Giza 12566, Egypt

**Keywords:** Meckel's diverticulum, Acute appendicitis, Gastrointestinal anomalies, Laparoscopic appendectomy, Pediatric surgery, Case report

## Abstract

Meckel's diverticulum (MD), a congenital abnormality occurring in 0.3% to 3% of individuals, shares overlapping clinical features with acute appendicitis (AA), a common surgical emergency, making simultaneous presentation diagnostically challenging. An 11-year-old boy presented with right lower abdominal pain, fever, and loss of appetite, exhibiting positive Rovsing and Psoas signs. Elevated inflammatory markers and ultrasound findings suggested acute appendicitis with concurrent Meckel's diverticulitis. Surgery confirmed acute suppurative appendicitis and an inflamed MD, both of which were successfully treated with appendectomy and segmental intestinal resection. The patient had an uneventful recovery and remained well during follow-up. This case highlights the importance of considering MD in pediatric abdominal pain and performing thorough intraoperative assessments to ensure accurate diagnosis and effective treatment.

## Introduction

Meckel's diverticulum (MD) and acute appendicitis (AA) rare encounters lead to a compelling medical dilemma as they are on opposing ends when it comes to gastrointestinal anomalies. Although MD is the most common gastrointestinal tract anomaly, it is very rare, only occurring in 0.3% to 3% of the general population [1]. It arises from the continuance of the omphalomesenteric duct, which is then obliterated during the second month of pregnancy, leading full layers of the small intestine covered by some gastric mucosa to form a diverticulum [2]. Only 2% of the cases are symptomatic and discovered in pediatric patients, predominantly those less than 24 months old, while the majority are asymptomatic [2]. The most common complication for MD is intestinal obstruction [1]. MD is mostly discovered during the management of other pathologies or routine imaging. MD often presents with acute abdominal symptoms, posing a diagnostic challenge due to its clinical similarity to other intra-abdominal inflammatory conditions. It is commonly mistaken for acute appendicitis, colonic diverticulitis, or inflammatory bowel disease. A preoperative diagnosis of MD is rare, occurring in fewer than 10% of cases involving a MD, with acute appendicitis being the most common preoperative diagnosis. (X) MD and AA are both predominant in the male sex [1,3]. On the other hand, AA is a common surgical emergency, with a prevalence of 8.3% in males. The Surgical CAse REport (SCARE) guideline criteria 2023 have been followed in the submission of this case report [4]. The aim of this case report is to embark on a journey through the fascinating intersection of these 2 conditions, shedding light on the complexities of diagnosis, treatment, and the pursuit of better patient care.

## Case presentation

**Fig. 1:**
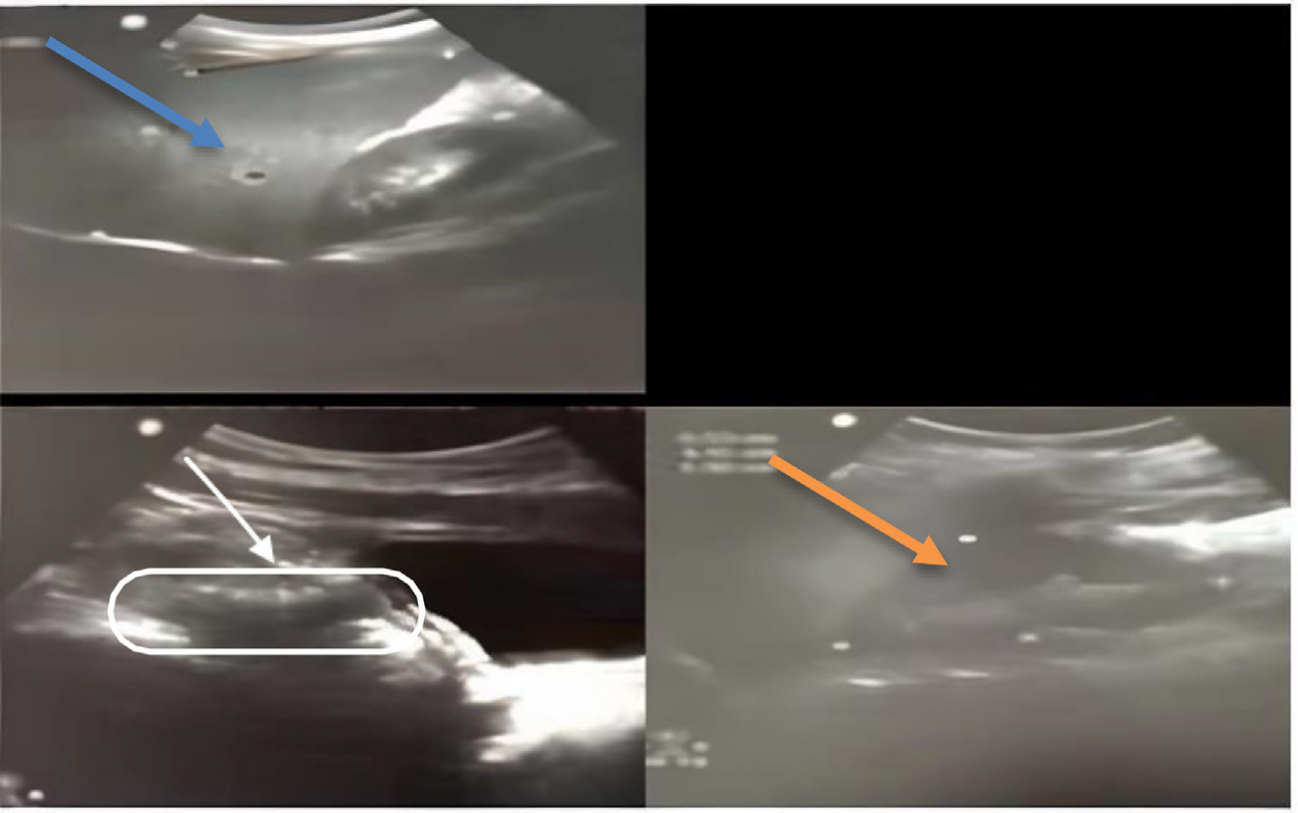
Pelviabdominal ultrasonography showing an peristaltic bowel loop in the right iliac fossa with distended distal part reaching about 7.5 mm and collapsed proximal half with echogenic wall till its origin and its origin seen inferior to the right of the umbilicus. A Minimal rim of fluid is seen around the peristaltic bowel loop and colonic gas distention is apparent.

**Fig. 2:**
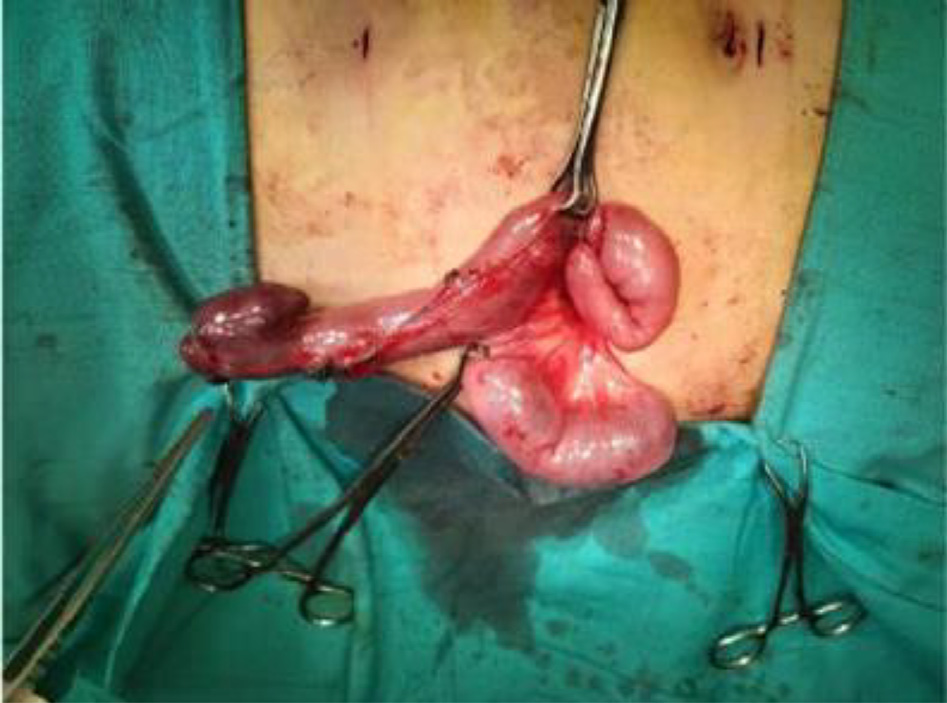
Intraoperative view of a 10 cm long intestinal loop with a red-black hemorrhagic clots and devitalized mucosal wall (an acute diverticulitis).

An 11-year-old male patient presented to the emergency department with a 1-day history of severe right lower quadrant abdominal pain, nausea, anorexia, and fever but no vomiting or diarrhea. There was no relevant medical history or previous surgery. Family history was irrelevant. A general examination revealed a fever of 38°C, tachycardia with normal blood pressure, no jaundice, no pallor, no cyanosis, and no lower limb edema. By local examination the abdomen was mildly distended, bowel sounds were hyperactive, with diffuse lower abdominal tenderness palpating mainly to the suprapubic and right iliac fossa areas, rebound tenderness, shifting tenderness, and Rovsing and Psoas signs both positive. The differential diagnosis includes Acute appendicitis, Terminal ileitis, Infective terminal ileitis, Omental infarction, Intestinal obstruction, diverticulitis, and Inflammatory bowel disease. Laboratory tests revealed inflammatory markers such as leukocytosis (white blood cells = 14.8 × 10^3^/ul) with neutrophilia (neutrophils = 82%), lymphopenia (lymphocytes =12%), and elevated C-reactive protein (CRP = 87 mg/dl). Urgent pelviabdominal ultrasonography (US) showed an aperistaltic bowel loop in the right iliac fossa with mild inflammation of the appendix and inflammation of the diverticulum (Fig. 1). patient was admitted to the ward and prepared for the surgery, In the operating room, diagnostic laparoscopy confirmed acute suppurative appendicitis and Meckel's diverticulitis of 10 cm in length with a “drumstick” tip (Fig. 2). The wide base mimics the diameter of the ileum, inflamed, causing hyperemia in the surrounding intestinal parts with prominent serosal vasculature. Instant, easy bleeding on touch was found at 110 cm from the ileocecal valve with a prominent edematous mesodiverticular band, and a mild, free pelvic reactionary collection was noted. Laparoscopic appendectomy, a limited side-to-side small bowel resection, and anastomosis in 2 layers were done simultaneously. Histopathological examination revealed Meckel's diverticulitis with inflammation and acute suppurative appendicitis. The patient had an unremarkable recovery in the hospital and remained well on outpatient follow-up a month later.

## Discussion

MD is a rare disease [1]. Despite being one of the most common gastrointestinal anomalies, asymptomatic and symptomatic patients are usually pediatric. Of those symptomatic patients, obstruction, hemorrhage, and infection are the most common presenting symptoms, respectively [1]. When MD is symptomatic, resection is the treatment of choice, and for accidental MDs, although there is still no agreement on the best management option, it seems sensible that risk factors act as a guide in making the decision [[5], [6], [7]]. The risk factors include patients below the age of 50 years, male patients, MD length > 2 cm, and ectopic or abnormal characteristics of a diverticulum, all of which are linked with symptomatic diverticulum and, therefore, would support resection upon detection argument [5]. For asymptomatic patients, surgical resection is not recommended for individuals of any age group due to retained MD having a lower risk of complications than open laparotomy, but it is important to note that its applicability to recent surgical practices involving laparoscopy remains uncertain [5]. MD and AA rare encounters lead to a fascinating medical dilemma in the literature, and clinically, it is an uphill battle to differentiate them using either insufficiently accurate radiological tests or clinical examination only. Hence, it is imperative not to approach the surgical procedure for acute appendicitis with a narrow perspective but, instead, to diligently explore for any concealed gastrointestinal anomalies. Complicated MD resection is necessary, and in cases of accidental MD, the risk factors should be used as guidance for resection [5].

## Conclusion

Despite the fact that we were able to do a laparoscopic resection of MD based on the patient risk factors with no complication, more research is needed to explore laparoscopic resection of asymptomatic accidental MDs benefit and although we had the knowledge of MD presence through US, The fact remains that imaging is a long way from being useful in these rare cases, therefore, the surgeon should always diligently explore for any concealed gastrointestinal anomalies, thereby ensuring a comprehensive evaluation during the operation.

## Patient consent

Written informed consent was obtained from the patient family for publication of this case report and accompanying images. A copy of the written consent is available for review by the Editor-in-Chief of this journal on request.
